# Acetic acid, growth rate, and mass transfer govern shifts in CO metabolism of *Clostridium autoethanogenum*

**DOI:** 10.1007/s00253-023-12670-6

**Published:** 2023-07-06

**Authors:** Marina P. Elisiário, Wouter Van Hecke, Heleen De Wever, Henk Noorman, Adrie J. J. Straathof

**Affiliations:** 1 Department of Biotechnology, Delft University of Technology, Van Der Maasweg 9, 2629HZ Delft, The Netherlands; 2 Flemish Institute for Technological Research (VITO), Boeretang 200, 2400 Mol, Belgium; 3 Royal DSM, Alexander Fleminglaan 1, 2613 AX Delft, The Netherlands

**Keywords:** CO metabolism, *Clostridium* autoethanogenum, Acetic acid concentration, Growth rate, Mass transfer

## Abstract

**Abstract:**

Syngas fermentation is a leading microbial process for the conversion of carbon monoxide, carbon dioxide, and hydrogen to valuable biochemicals. *Clostridium autoethanogenum* stands as a model organism for this process, showcasing its ability to convert syngas into ethanol industrially with simultaneous fixation of carbon and reduction of greenhouse gas emissions. A deep understanding on the metabolism of this microorganism and the influence of operational conditions on fermentation performance is key to advance the technology and enhancement of production yields. In this work, we studied the individual impact of acetic acid concentration, growth rate, and mass transfer rate on metabolic shifts, product titres, and rates in CO fermentation by *C. autoethanogenum*. Through continuous fermentations performed at a low mass transfer rate, we measured the production of formate in addition to acetate and ethanol. We hypothesise that low mass transfer results in low CO concentrations, leading to reduced activity of the Wood–Ljungdahl pathway and a bottleneck in formate conversion, thereby resulting in the accumulation of formate. The supplementation of the medium with exogenous acetate revealed that undissociated acetic acid concentration increases and governs ethanol yield and production rates, assumedly to counteract the inhibition by undissociated acetic acid. Since acetic acid concentration is determined by growth rate (via dilution rate), mass transfer rate, and working pH, these variables jointly determine ethanol production rates. These findings have significant implications for process optimisation as targeting an optimal undissociated acetic acid concentration can shift metabolism towards ethanol production.

**Key points:**

• *Very low CO mass transfer rate leads to leaking of intermediate metabolite formate.*

• *Undissociated acetic acid concentration governs ethanol yield on CO and productivity.*

• *Impact of growth rate, mass transfer rate, and pH were considered jointly.*

**Graphical abstract:**

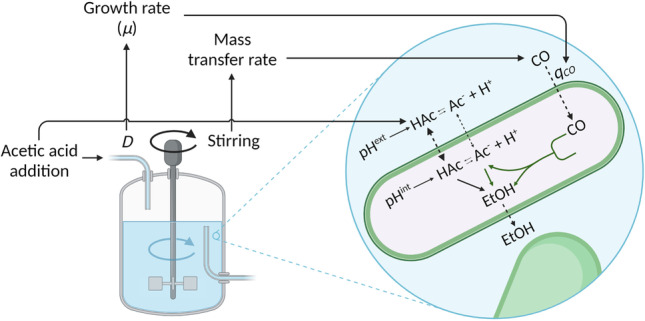

## Introduction

**Fig. 1:**
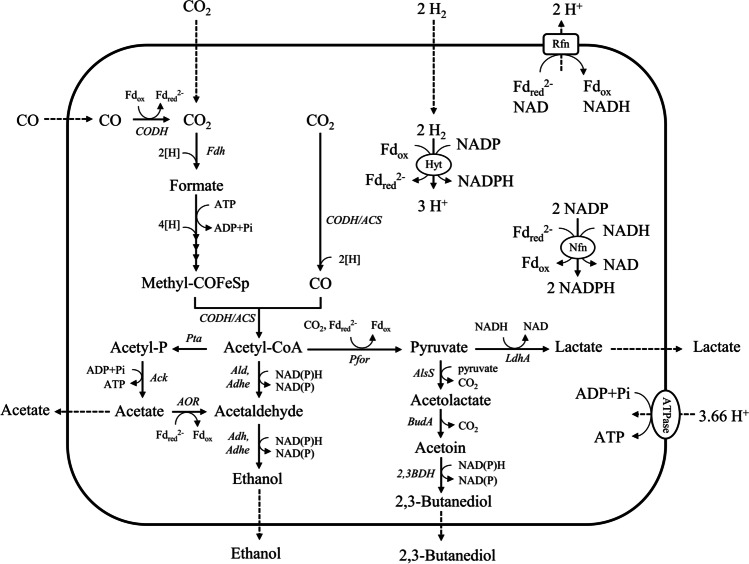
Simplified overview of carbon fixation through WLP and autotrophic product formation in *C. autoethanogenum,* including key enzymes and product excretion. Figure adapted from Liew et al. (2016) and Liew et al. (2017), wherein abbreviations are depicted

Syngas fermentation is a microbial process through which acetogenic microorganisms convert carbon monoxide (CO), carbon dioxide (CO_2_), and hydrogen (H_2_) into added-value biochemical compounds (Abubackar et al. 2011; Phillips et al. 2017). This technology offers a ground-breaking option for green-house gas emission reduction and sustainable biochemical production (Liew et al. 2016). The acetogen *Clostridium autoethanogenum* is a syngas-fermenting model organism, which can natively produce ethanol (EtOH), acetate (Ac), 2,3-butanediol (BDO), and lactate (Köpke et al. 2011); therefore, it has been vastly studied and is employed for industrial production of ethanol (Abrini et al. 1994; Bengelsdorf and Dürre 2017; Bengelsdorf et al. 2018). This microorganism uses the Wood–Ljungdahl pathway (WLP) for reductive synthesis of acetyl-CoA from CO_2_, CO, and H_2_ (Fig. 1).

Understanding the metabolism of this microorganism and the impact of fermentation conditions on fermentation performance is key to advance the technology. Among other factors, the gas-to-liquid mass transfer rate has a very pertinent role, as it directly affects the substrate availability to the microorganism (Elisiário et al. 2022). For example, the substrate uptake rate depends on the dissolved gas concentration. Different substrate gas-to-liquid mass transfer rates can be imposed, for example, by changing the agitation rate in stirred tank reactors or the superficial gas velocity in stirred tank and bubble column reactors (Asimakopoulos et al. 2018; Elisiário et al. 2022).

In addition to the gas-to-liquid mass transfer rate, the biomass-specific microbial growth rate, *μ,* also has a crucial role in the fermentation performance (de Lima et al. 2022). In chemostat fermentations, the biomass-specific growth rate *μ* is equivalent to and imposed by the operational dilution rate *D*, up to the critical dilution rate. The growth rate influences the product concentration and substrate to product yields. Increasing the growth rate might also change product distribution because the microbe may shift its metabolism (Heffernan et al. 2020; de Lima et al. 2022) to cope with higher energy requirements for biomass formation. Process rates are affected, which has an impact on process economics.

The combined influence of growth rate and mass transfer rate also determines the production rate and concentration of total acetate (anion plus undissociated species). Simultaneously, the extracellular pH determines the extracellular ratio of acetate anion to undissociated acetic acid, which can differ from the intracellular ratio (Richter et al. 2016) since the intracellular pH is metabolically controlled at about 6.0 (Mock et al. 2015) and the external pH can be imposed.

Since many factors determine the syngas fermentation performance (microorganism, pH, medium composition, gas flow, gas composition, dilution rate, etc.) and literature covers only part of the operational window, understanding of the impact of operational settings on the fermentation performance is still incomplete.

In this work, we will obtain and characterise chemostat fermentations of *C. autoethanogenum* grown on CO as sole carbon and energy source, to simplify the system. We will investigate the individual effects of CO mass transfer rate (100 or 500 rpm agitation in a stirred bioreactor), growth rate (~ 0.008 h^−1^ to ~ 0.04 h^−1^ dilution rate), and acetate concentration on products distribution, titres, rates, and yields. An assessment will be made of how the individual effects jointly affect the observed fermentation performance and shifts in the metabolic network (Fig. 1). Previous studies have hypothesised on the role of acetic acid as a critical factor in increasing ethanol production in syngas fermentation (Richter et al. 2016; Valgepea et al. 2017). We will further provide experimental evidence to support this hypothesis for *C. autoethanogenum*, by analysing experiments supplementing exogenous acetic acid to fermentation while keeping other fermentation conditions constant. Furthermore, we will conduct a comprehensive analysis and comparison of literature experimental results to clarify the role of acetic acid concentration on ethanol yield.

## Materials and methods

### Microorganism, growth medium, and inoculum cultivation

*C. autoethanogenum* (DSM 10061) from the DSMZ strain collection (Braunschweig, Germany) was used in all fermentations and stored as glycerol stock at − 80 °C. Pre-cultures were cultivated in batch operation in anaerobic bottles capped with rubber stoppers and aluminium caps (50 mL working volume), at 37 °C without agitation, after inoculation in a 1:50 ratio (v/v). The glycerol stock cells were first revived in modified YTF (yeast extract-tryptone-fructose) medium (containing per litre: 10 g Bacto™ yeast extract, 16 g tryptone, 4 g NaCl, 4 mg Cl_2_Fe ⋅ 4 H_2_O, 0.5 mg resazurin sodium salt, and 0.75 g L-cysteine · HCl · H_2_O dissolved in demineralised water) adjusted to pH 6.2 with 2 mol L^−1^ HCl and under 100% N_2_ headspace (1.5 atm). Once this culture reached exponential growth, the cells were propagated and further cultivated under 100% CO headspace (1.5 atm) in anaerobic bottles with the feed medium. Once exponentially growing, this culture was used as inoculum for bioreactor experiments, in a 1:20 v/v ratio. The feed medium contained per litre: 0.9 g NH_4_Cl, 0.9 g NaCl, 0.2 g MgSO_4_ · 7 H_2_O, 0.7 g KH_2_PO_4_, 1.5 g K_2_HPO_4_, 0.02 g CaCl_2_, 0.5 mg resazurin sodium salt, 0.5 g Bacto™ yeast extract, and 0.75 g L-cysteine · HCl · H_2_O dissolved in demineralised water; and it was supplemented with the following metal trace elements per litre of medium: 1.5 mg FeCl_2_ · 4 H_2_O, 2.5 mg FeCl_3_ · 6 H_2_O, 0.07 mg ZnCl_2_, 0.1 mg MnCl_2_ · 4 H_2_O, 0.006 mg H_3_BO_3_, 0.19 mg CoCl_2_ · 6 H_2_O, 0.002 mg CuCl_2_ · 2 H_2_O, 0.024 mg NiCl_2_ · 6 H_2_O and 0.04 mg Na_2_MoO_4_ · 2 H_2_O, 0.004 mg Na_2_SeO_3_, and 0.2 mg Na_2_WO_4_ · 2 H_2_O. Additionally, the feed medium contained the following vitamins per litre of medium: 0.02 mg biotin, 0.2 mg nicotinamide, 0.1 mg p-aminobenzoic acid, 0.2 mg thiamine · HCl, 0.1 mg pantothenic acid, 0.5 mg pyridoxamine, 0.1 mg cyanocobalamin, and 0.1 mg riboflavin. The pH of the feed medium was adjusted to 6.2 with 2 mol L^−1^ HCl. Both media were sterilised by autoclaving at 121 °C during 20 min. The yeast extract, vitamins, and cysteine were added to the media as sterile concentrated stock solutions after autoclavation. The feed medium for the steady-state fermentation VI is additionally supplemented with a concentrated sterile acetic acid solution to reach the concentration mentioned in Table 1.

**Table 1:** Operational conditions of fermentations at steady state

Steady state	I	II	III	IV	V	VI
Working volume (L)	0.9	0.9	1.0	1.0	1.0	1.0
Stirring rate (rpm)	100	100	500	500	500	500
Inlet CO concentration (%)	40	40	50	50	50	50
Dilution rate (h^−1^)	0.0081 ± 0.0004	0.025 ± 0.001	0.0088 ± 0.0004	0.024 ± 0.001	0.039 ± 0.002	0.040 ± 0.002
Total acetate concentration in the feed media (g L^−1^)	0	0	0	0	0	10.15 ± 0.11

### Bioreactor operation

Continuous fermentations for cultivation of *C. autoethanogenum* were performed in a 1.5-L glass jacketed stirred tank bioreactor (Applikon, Delft, The Netherlands). Three baffles and two Rushton impellers (46 mm diameter) were installed; the impellers were placed at 33% and 66% of the liquid height. The fermentation pH, temperature, agitation, and mass flow were controlled (In-Control, Applikon, The Netherlands). Conditions were strictly anaerobic at 37 °C. Off-gas was condensed at 4 °C, such that water and ethanol loss was insignificant. The pH of the fermentation was maintained at 5.90 ± 0.05 by addition of 2 mol L^−1^ NaOH via a peristaltic pump. The start-up, inoculation, and batch operation of the bioreactor were performed as reported by Diender et al. (2019). Peristaltic pumps (Masterflex, Gelsenkirchen, Germany) were used for continuous supply of feed medium and removal of effluent, applying different dilution rates (Table 1). The bioreactor was continuously supplied with a gas phase of 10 mL min^−1^ (on basis of standard temperature and pressure) consisting of CO and N_2_ (composition in Table 1). Variable stirring rates were applied. Effluent samples of 2 mL were analysed daily for biomass concentration using optical density. Each sample supernatant was analysed for product concentration using ultra performance liquid chromatography (UPLC). Off-gas composition was continuously monitored. The steady-state (SSt) results were obtained from three independent chemostat runs and were reported once concentrations were constant for at least 3 working volume changes.

### Analytical techniques

Optical density of broth was measured daily at 660 nm (OD_660_). When constant, the biomass concentration was measured (at least in triplicate) by determination of the volatile suspended solids (VSS) concentration in the broth (Clesceri et al. 1999), from 150 mL broth samples collected continuously and anaerobically from the effluent of the bioreactor.

Acetate, ethanol, 2,3-butanediol, and formate concentrations in filtered broth samples (0.22-µm pore size, Millipore, Millex-GV, MA, USA) were determined using ultra high-performance liquid chromatography (UPLC) with an Aminex HPX-87 H column (BioRad, CA, USA) and 1.5 mmol L^−1^ phosphoric acid as eluent at 50 °C with RI detection (RefractoMax 520, Thermo Fisher Scientific, MA, USA).

The bioreactor exhaust gas was continuously diluted 1:10 (v/v) with pure nitrogen gas to obtain the minimum flow required for gas analysis (Rosemount™ X-STREAM XEGP, Emerson, MO, USA). This custom-built analyser was equipped with a nondispersive infrared (NDIR) sensors for CO and CO_2_ measurement and a thermal conductivity detector (TCD) for H_2_ measurement.

### Quantification of fermentation data

#### Production rates

Production rates, ${R}_{i}$ (mmol h^−1^), were quantified for analysis of fermentation performance. The off-gas flow rate, ${F}_{n}^{G,out}$, was calculated using an N_2_ (inert) gas mass balance with its concentrations measured in the gas inlet and outlet. The production rates were calculated from compound mass balances, taking into account gas phase inlet and outlet molar fractions, ${x}_{i}^{G}$, and molar flow rates, ${F}_{n}^{G}$, for gaseous products (CO_2_, CO and H_2_) (Eq. 1) and considering liquid outlet product concentration, ${C}_{i}^{L, out}$, and volumetric flow rate, ${F}_{L}^{out}$, for aqueous products (Ac, EtOH, formate, and biomass) (Eq. 2).

1 $$R_{i} = x_{i}^{G,out} F_{n}^{G,out} - x_{i}^{G,n} F_{n}^{G,in}$$

2 $$R_{i} = C_{i}^{L,out} F_{L}^{out}$$

The biomass-specific production rates *q*_*i*_ (mmol g_X_^−1^ h^−1^) were calculated from *R*_*i*_, liquid working volume, *V*_*L*_, and biomass concentration *c*_*X*_ using Eq. 3.

3 $$q_{i} = \frac{{R_{i} }}{{c_{X} . V_{L} }}$$

#### Carbon and electron balances

Fermentation data analysis and reconciliation were performed using carbon and electron balances.

Carbon recoveries were calculated from production rate *R*_*i*_ (mol h^−1^) per compound *i* (positive or negative), with its number of carbon atoms *n*_*C,i*_ (mol_C_ mol_i_^−1^) (see Eq. 4). Electron recoveries were calculated from *R*_*i*_ and the degree of reduction *γ*_*i*_ (mol_e_ mol_i_^−1^) (see Eq. 5). The reference degrees of reduction were: C = 4, H = 1, N =  − 3, O =  − 2, (^+^) =  − 1, and (^−^) = 1. For these calculations, CO was considered to be the sole carbon source and electron donor. The biomass (*X*) composition was assumed to be CH_1.8_O_0.5_N_0.2_ (Heijnen and Kleerebezem 2010), resulting in 24.6 mol_*x*_ g_*x*_^−1^.

4 $$C_{rec} = \frac{{\mathop \sum \nolimits_{i}^{n} R_{i} .n_{C,i} }}{{ - R_{CO} . n_{C, CO} }}; \, i = \left\{ {\left. {{\text{CO}}_{{2}} {\text{, Ac, EtOH, BDO, formate, X}}} \right\}} \right.$$

5 $$e_{rec} = \frac{{\mathop \sum \nolimits_{i}^{n} R_{i} .\gamma_{i} }}{{ - R_{CO} . \gamma_{CO} }}; \, i = \left\{ {\left. {{\text{H}}_{{2}} {\text{, Ac, EtOH, BDO, formate, X}}} \right\}} \right.$$

## Results

### Comparison of different mass transfer rates

We compared the impact of agitation rate (100 or 500 rpm) at two fixed growth rates (~ 0.009 h^−1^ and ~ 0.024 h^−1^; steady states I to IV in Table 1). This 5 × rise in agitation speed corresponds to a 125 × increase in the power input per volume (P/V), considering properly operating impellers in the turbulent flow regime and for coalescing broth. Subsequently, the corresponding volumetric mass transfer coefficient is expected to increase 6 to 7 times, according to van’t Riet (1979), and hence, the CO transfer capacity as well. The observed mass transfer rate of CO and the associated CO consumption rate were expected to increase by such a factor only in case of mass transfer limitation occurring at either stirring rate. Higher agitation indeed led to an increase of CO consumption from about 2 mmol L^−1^ h^−1^ (estimated from production rates and catabolic stoichiometries) to 10 mmol L^−1^ h^−1^ (measured experimentally), which indicates mass transfer limitation at these conditions. For growth rates of ~ 0.009 and ~ 0.024 h^−1^, the biomass concentration increased 6 and 7 times, respectively.

**Fig. 2:**
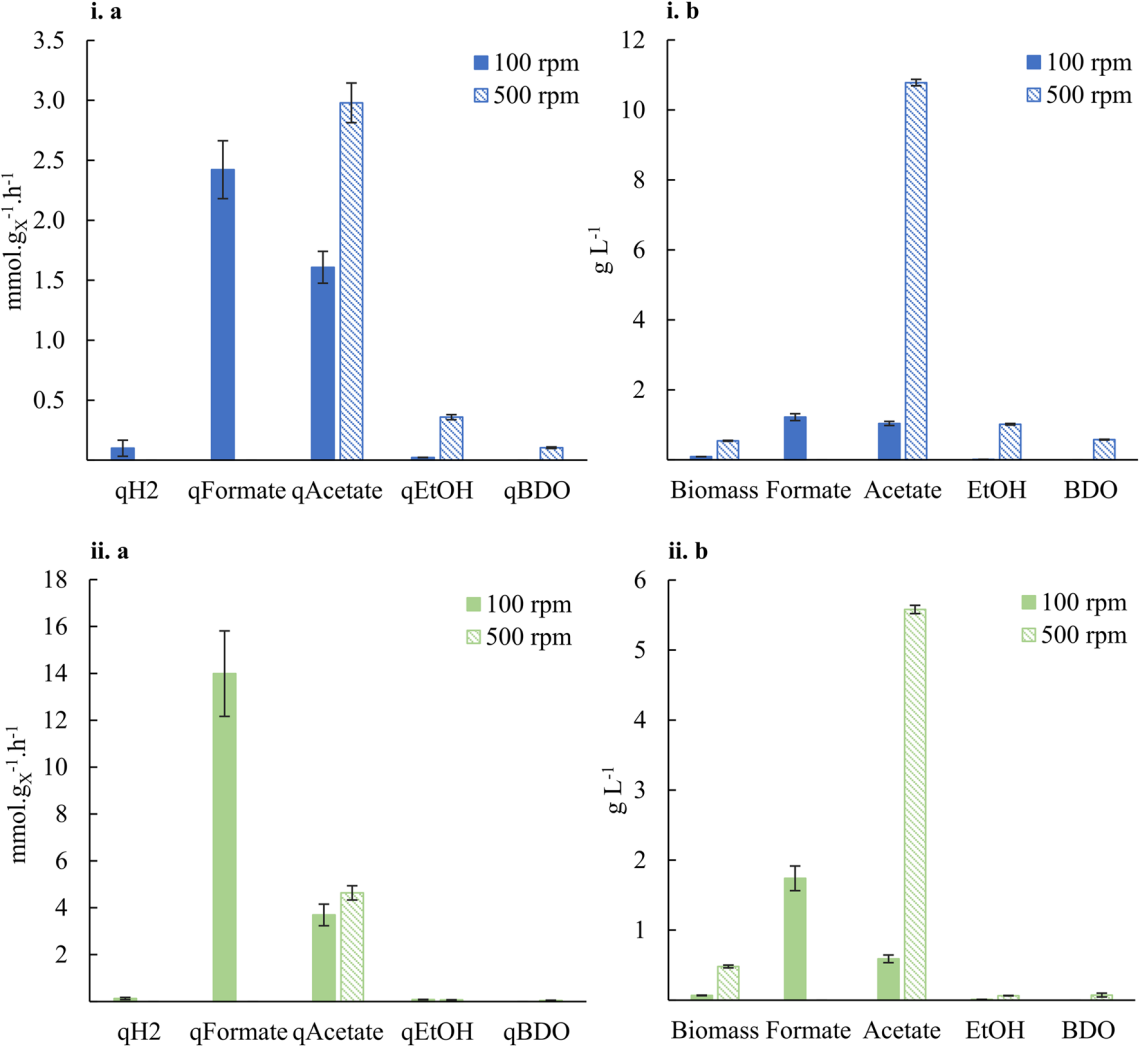
Biomass specific production rates (**a**) and product concentrations (**b**) for steady-state fermentations grown at ~ 0.009 h^−1^ (i) and ~ 0.024 h^−1^ (ii)

Figure 2 shows that the fermentations at 500 rpm led to the expected products (acetate, ethanol, and 2,3-butanediol), with relatively high titres and biomass-specific production rates for acetate and ethanol. However, for the fermentations at 100 rpm, production of formate was substantial (14.6% and 41.4% of the converted carbon, for SSt I and II, respectively).

**Fig. 3:**
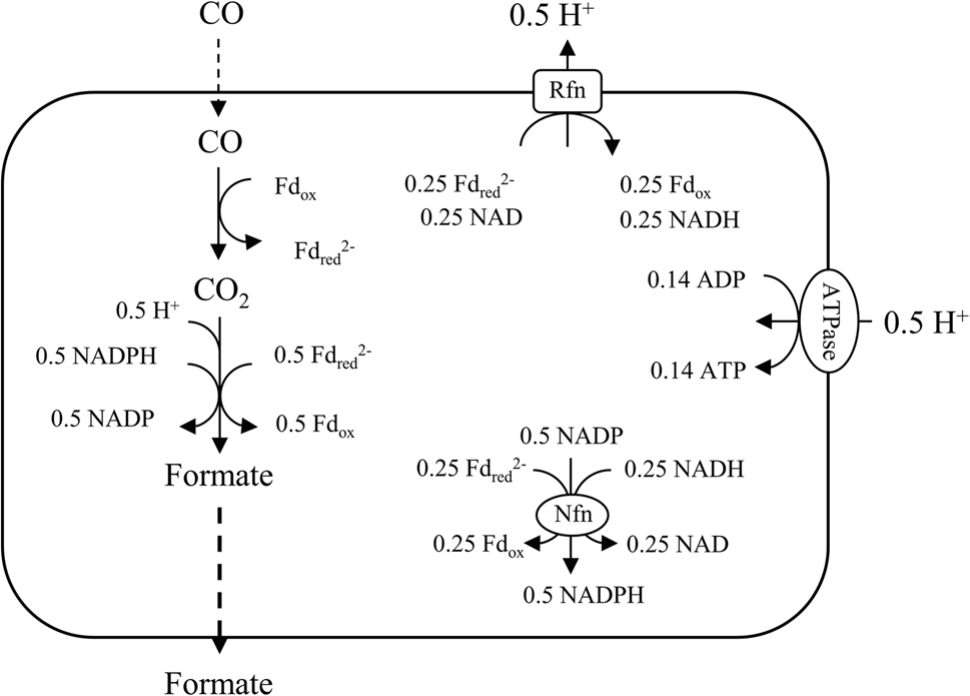
Schematic overview of the proposed pathway for CO conversion to formate with ATP production in *C. autoethanogenum*

In Fig. 3, we propose a combination of reactions that allows the microorganism to gain some adenosine triphosphate (ATP) from CO conversion into formate (0.14 mol_ATP_/mol_CO_), in case no ATP is required for formate export. The yield of ATP is significantly higher (0.375 mol_ATP_/mol_CO_) if acetate would be produced (Allaart et al. 2023). A reason for the organism to excrete formate instead of converting it into acetate could be severe limitation of CO, of which a second molecule is required per acetyl-CoA and hence acetate (see Fig. 1). As explained before, at 100 rpm agitation rate, mass transfer limitation is severe, so dissolved CO concentrations can be assumed to be very low, but quantification would require specific equipment (Mann et al. 2021) or methods for reliable *k*_L_*a* determination in the presence of broth components (Puiman et al. 2022).

### Comparison of different growth rates

**Fig. 4:**
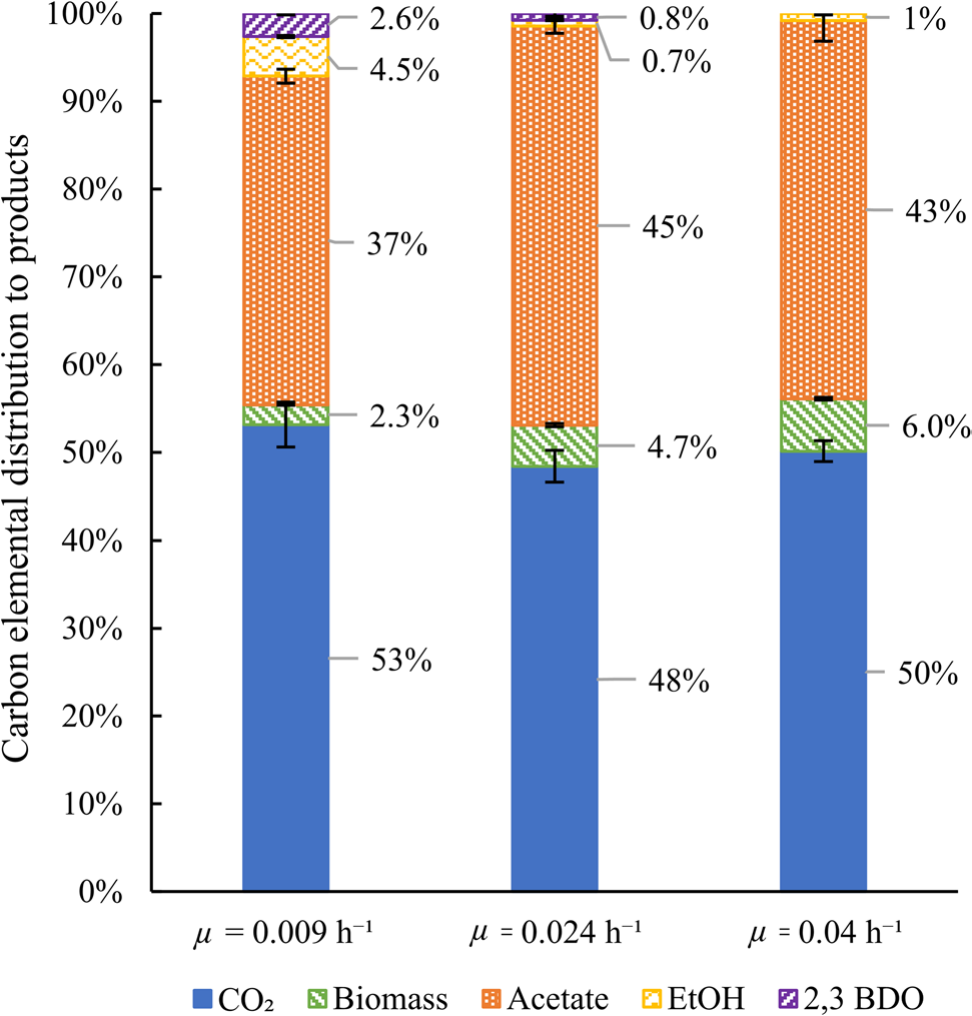
Carbon distribution from CO to products in steady-state fermentations at 500 rpm stirring rate. Carbon recoveries were normalised to 100% to facilitate comparison of carbon distributions between different conditions

At fixed agitation rate and volumetric CO supply rate, we tested different growth rates by changing the dilution rate accordingly (steady states III, IV, and V in Table 1). For these experiments, correct gas measurements were available, and the carbon and electron recoveries indicate highly consistent data. Namely, for *μ* = 0.009, 0.024, and 0.04 h^−1^, carbon recoveries (Eq. 4) were 95 ± 2%, 94 ± 1%, and 98 ± 1%, respectively, and electron recoveries (Eq. 5) were 95 ± 2%, 99 ± 2%, and 99 ± 5%, respectively. Fig. 4 shows the carbon distribution, indicating acetate and CO_2_ as main products.

**Fig. 5:**
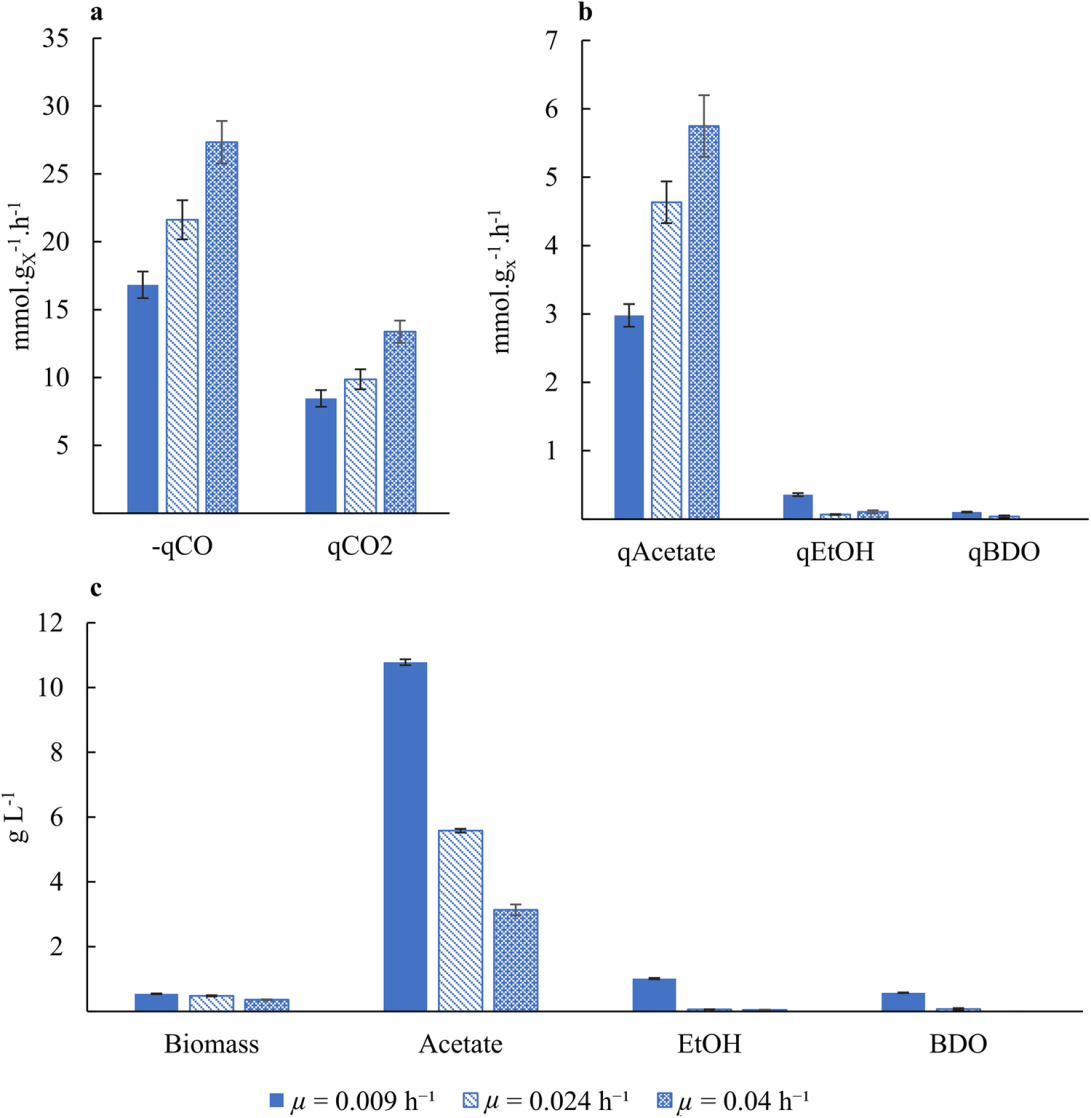
Continuous fermentation of *C. autoethanogenum* grown at 500 rpm stirring rate (steady states III-IV-V corresponding to *μ* = 0.009–0.024–0.04 h^−1^, respectively). **a** and **b** Biomass-specific production rates. **c** Product concentrations

As expected in a substrate limited regime, an increase of *µ* results in a linear increase of − *q*_CO_ as shown in Fig. 5. The higher − *q*_CO_ value correlates with an increase in *q*_Ac_ and *q*_CO2_ as the catabolic reaction to acetate and CO_2_ generates most ATP for biomass production. On the other hand, *q*_EtOH_ and *q*_BDO_ generally decreased with increasing *µ*. Our results also show that, at the studied fermentation settings, faster growth rates do not result in increased production rates of the more reduced products (ethanol and BDO). This is supported by Fig. 5c, where an increase of *µ* leads to lower ethanol and BDO concentrations. Also, the acetate concentration in broth decreases.

### Influence of acetate addition

For *µ* = 0.04 h^−1^ at 500 rpm agitation rate, the impact of extracellular acetate (and consequently undissociated acetic acid) on ethanol yield and productivity was studied by supplementing acetate to feed medium at fixed pH. The operating conditions for the obtained steady states V and VI are given in Table 1, and the results are compared in Table 2.

**Table 2:** Comparison of steady-state conditions and conversion between experiments with (SSt VI) and without (SSt V) acetate in the feed medium

Steady-state fermentation	V	VI
Volume-specific acetate feed rate (g L^−1^ h^−1^)	0	0.408 ± 0.004
Growth rate (h^−1^)	0.039 ± 0.002	0.040 ± 0.002
Biomass concentration (g L^−1^)	0.36 ± 0.01	0.26 ± 0.01
Acetate concentration (g L^−1^)	3.13 ± 0.17	11.38 ± 0.11^a^
EtOH concentration (g L^−1^)	0.045 ± 0.009	0.318 ± 0.006
BDO concentration (g L^−1^)	0	0
*q*_CO_ (mmol g_x_ L^−1^)	− 27.3 ± 1.6	− 31 ± 2
*q*_CO2_ (mmol g_x_ L^−1^)	13.4 ± 0.8	16.4 ± 1.6
*q*_Ac_ (mmol g_x_ L^−1^)	5.8 ± 0.5	3.2 ± 0.3
*q*_EtOH_ (mmol g_x_ L^−1^)	0.11 ± 0.02	1.05 ± 0.07
*Y*_EtOH/CO_ (mol_EtOH_ mol_CO_^−1^)	0.0039 ± 0.0008	0.0340 ± 0.0009

^a^Substracting the added acetate leads to 1.23 ± 0.11 g L^−1^ produced acetate

For SSt VI, *q*_Ac_ was significantly lower than for SSt V, where total acetate concentration (resulting only from microbial production) was much lower. On the other hand, *q*_EtOH_ and yield of ethanol on CO, *Y*_EtOH/CO_, increased both almost tenfold at the increased acetate concentration. This coincided with an increase of 13.5% in − *q*_CO_ and 22.4% in *q*_CO2_ and a decrease of 27.8% in biomass concentration. BDO production was not measured upon the addition of acetate to the feed medium.

## Discussion

### Acetic acid increases ethanol yield on CO

The maximum amount of ATP produced for the catabolic conversion of CO to acetate and to ethanol is given by (Bertsch and Müller 2015; Allaart et al. 2023):

6 $${\text{4 CO + 2 H}}_{{2}} {\text{O }} \to {\text{ Ac + 2 CO}}_{{2}} { + 1}{\text{.5 ATP}}$$

7 $${\text{6 CO + 3 H}}_{{2}} {\text{O }} \to {\text{ EtOH + 4 CO}}_{{2}} { + }2.1{\text{ AT}}P$$

Per converted CO, more ATP is produced in case of acetate than in case of ethanol production. Therefore, acetate is the main product if CO is limiting, but still sufficiently available to prevent formate production. However, like Diender (2019), we found that addition to acetate to the feed resulted in decreased acetate production, increased volumetric ethanol production rates, and decreased biomass concentrations. Toxicity of undissociated acetic acid to the microorganism was proposed (Diender 2019). Similar results on ethanol productivity have been obtained by others with other microbial strains or other gas compositions (Gaddy et al. 2003; Kwon et al. 2022; Schulz et al. 2023).

The undissociated acetic acid concentration depends on pH and measured total acetate concentration (dissociated plus undissociated).

**Fig. 6:**
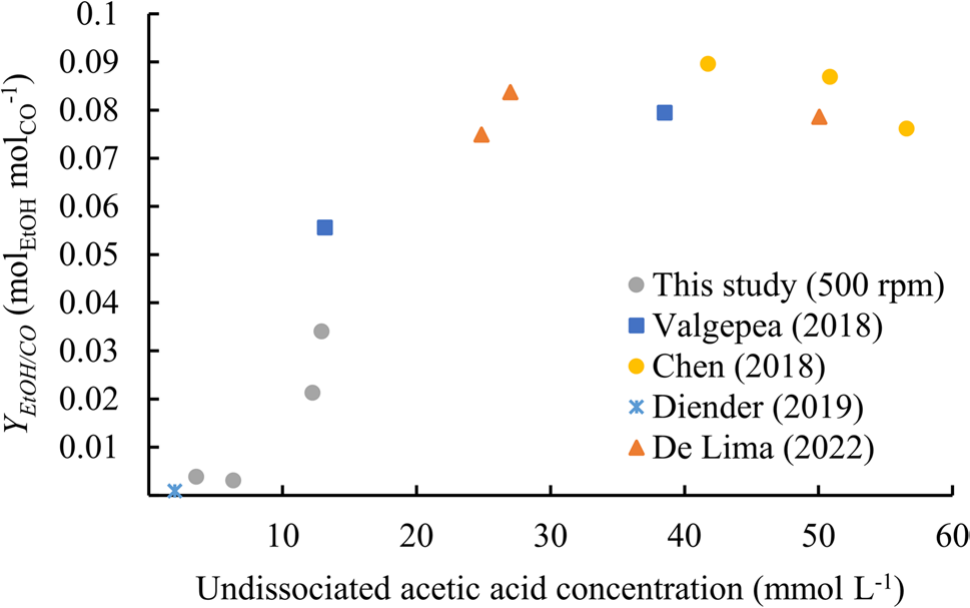
Ethanol yield on CO for different continuous fermentations of *C. autoethanogenum* grown on CO as function of the extracellular acetic acid concentration, which was calculated from the concentration of total acetate and pH at steady state

Figure 6 compares our results with literature data from CO fermentations by *C. autoethanogenum*. Despite different fermentation conditions among studies, the general trend is that ethanol yield on CO increases with increasing extracellular acetic acid concentration until a plateau is reached. While the maximum catabolic yield is 0.17 mol_EtOH_ mol_CO_^−1^ according to Eq. 7, the maximum experimental yields reported are close to 0.090 mol_EtOH_ mol_CO_^−1^ for acetic acid concentrations higher than 25 mmol L^−1^_._

At extracellular pH around 6.0, a small part of total acetate is protonated. The higher the extracellular total acetate concentration or the lower the pH, the higher is the resulting undissociated acetic acid concentration. Acetic acid has a significantly higher permeability coefficient than acetate (Valgepea et al. 2017), and therefore, it diffuses faster into the cell. When acetic acid diffuses back into the cell, it carries a proton that is not being imported through ATPase, therefore not producing ATP. This explains the inhibitory effect of acetic acid on the microorganism, as higher concentrations of acetic acid lead to the uncoupling of proton motive force and respective higher ATP maintenance requirements (Valgepea et al. 2017). As a strategy to restrict such ATP loss, the microorganism drives the metabolism towards acetate conversion to ethanol, even though *Y*_ATP/CO_ is lower when CO is overall converted into ethanol than in acetate (Eqs. 6 and 7). Similarly, for *Clostridium ljungdahlii*, Richter et al. (2016) have stated that at a thermodynamic threshold concentration of undissociated acetic acid with a surplus of reducing equivalents, ethanol production occurs as an overflow mechanism.

Comparably to our experimental results, Xu et al. (2020) demonstrated that ethanol production is increased (through acetate reduction via an aldehyde:ferredoxin oxidoreductase (AOR)), upon supplementation of additional extracellular (^13^C-labeled) acetate to the cultivation medium of *C. autoethanogenum*, growing on 100% CO in batch experiments, and posterior detection of ^13^C-labeled ethanol. The indirect ethanol pathway, through acetate reduction via AOR, has been postulated and discussed before (Köpke et al. 2010; Basen et al. 2014; Mock et al. 2015), and its role in autotrophic ethanol production in *C. autoethanogenum* has been confirmed by Liew et al. (2017).

Product inhibition in chemostats leads to lower biomass concentration (Straathof 2023), in line with our observation in Table 2. These results emphasise that acetic acid concentration is a key fermentation variable that determines metabolic shifts in CO-fermenting acetogenic bacteria *C. autoethanogenum*, and consequently, also impacts product distribution, ethanol yield and volumetric productivities. Since mass transfer rate and growth rate affect the resulting total extracellular acetate concentration, and pH the undissociated acetic acid concentration, these factors are equally fundamental to determine product distribution to ethanol (see subsequent sections). This finding has pertinent implications for industrial process operation and for tuning metabolic shifts towards solventogenesis. For example, by lowering operational pH or recovering and recycling acetate to the bioreactor, one could drive production towards higher ethanol yields and productivity.

### Influence of mass transfer

Decreasing the fermentation agitation rates corresponds to decreased gas-to-liquid mass transfer rates, dissolved CO concentration in the broth, and CO uptake rate. For *C. autoethanogenum* fermentations, we observed excretion the intermediate metabolite formate. Formate production in syngas fermentation has been previously observed during batch cultivation of *Clostridium ljungdahlii* (Oswald et al. 2018; Stoll et al. 2019) and in *Acetobacterium woodii* (Peters et al. 1999; Kantzow and Weuster-Botz 2016) and has been linked to high partial pressure of dissolved CO_2_ or H_2_. Our continuous fermentations have no CO_2_ or H_2_ feeding, and we expect very low CO concentrations when formate is excreted. Formate excretion might be caused by its accumulation due to a bottleneck in a consecutive conversion step in the WLP. Formate formation is the first step in the methyl branch, and reducing equivalents is required to convert it to methyl-COFeSp which is a substrate for CODH/ACS. In the case of low CO concentration conditions, there could be a deficiency of reducing equivalents, which are typically supplied by CO oxidation to CO_2_. The lack of reducing equivalents (due to severe limitation in CO availability) could potentially hinder the further conversion of formate to methyl-COFeSp, resulting in the accumulation of formate.

*C. autoethanogenum* harbours more than one CO dehydrogenase (CODH) enzyme, which can catalyse the reversible CO oxidation to CO_2_ (Liew et al. 2016) (Fig. 1). One of the CODHs combines with acetyl-CoA synthase (ACS) to form the bifunctional CODH/ACS complex for CO_2_ reduction to CO and acetyl-CoA fixation (Fig. 1). In the case that the activity for CO conversion would be much higher for the CODH which catalyses CO to CO_2_, than for ACS which catalyses CO to acetyl-CoA, formate accumulation could be explained. A reason for the relatively low activity of ACS complex in case of low CO concentration might be that the Michaelis constant of the CODH for CO is well below the CO concentration, in combination with a Michaelis constant of ACS for CO well above the CO concentration, such that only ACS loses activity. Enzyme affinities for CO will need to be measured to test this hypothesis, using, for example, approaches and methods similar to Techtmann et al. (2011).

Increasing mass transfer triggers other effects. Assuming a carbon-limited continuous cultivation, for a fixed growth rate, Valgepea et al. (2017) expected to obtain the same *q*_*CO*_ for different stirring rates (and corresponding substrate transfer and uptake rate, and biomass concentrations). Instead, Valgepea et al. (2018) observed an increase in − *q*_*CO*_ from ~ 22 to ~ 31 mmol g_X_^−1^ h^−1^ when increasing stirring rate from 510 to 650 rpm, in case of growing *C. autoethanogenum* solely on CO at *µ* =  ~ 0.04 h^−1^. Besides, increased agitation rate resulted in higher production of acetate and ethanol and a higher acetate to ethanol ratio.

We explain this as follows: For a fixed growth rate, an increase in agitation rates also translates into higher biomass concentration and *q*_*Ac*_ and consequently in higher acetic concentration. This is aligned with our experimental results (Fig. 2) and by Valgepea et al. (2018). Given the inhibitory effect of undissociated acetic concentration on the microorganism, this consequence elucidates the metabolic shifts and carbon distribution in *C. autoethanogenum*, when varying substrate mass transfer rate. It also explains the increase of − *q*_*CO*_ for higher mass transfer rates obtained by Valgepea et al. (2018) and the resulting increased acetic acid concentrations, since cells dissipate more CO as CO_2_ for maintenance and consequently a lower carbon fraction is allocated to biomass growth.

### Influence of growth rate

**Fig. 7:**
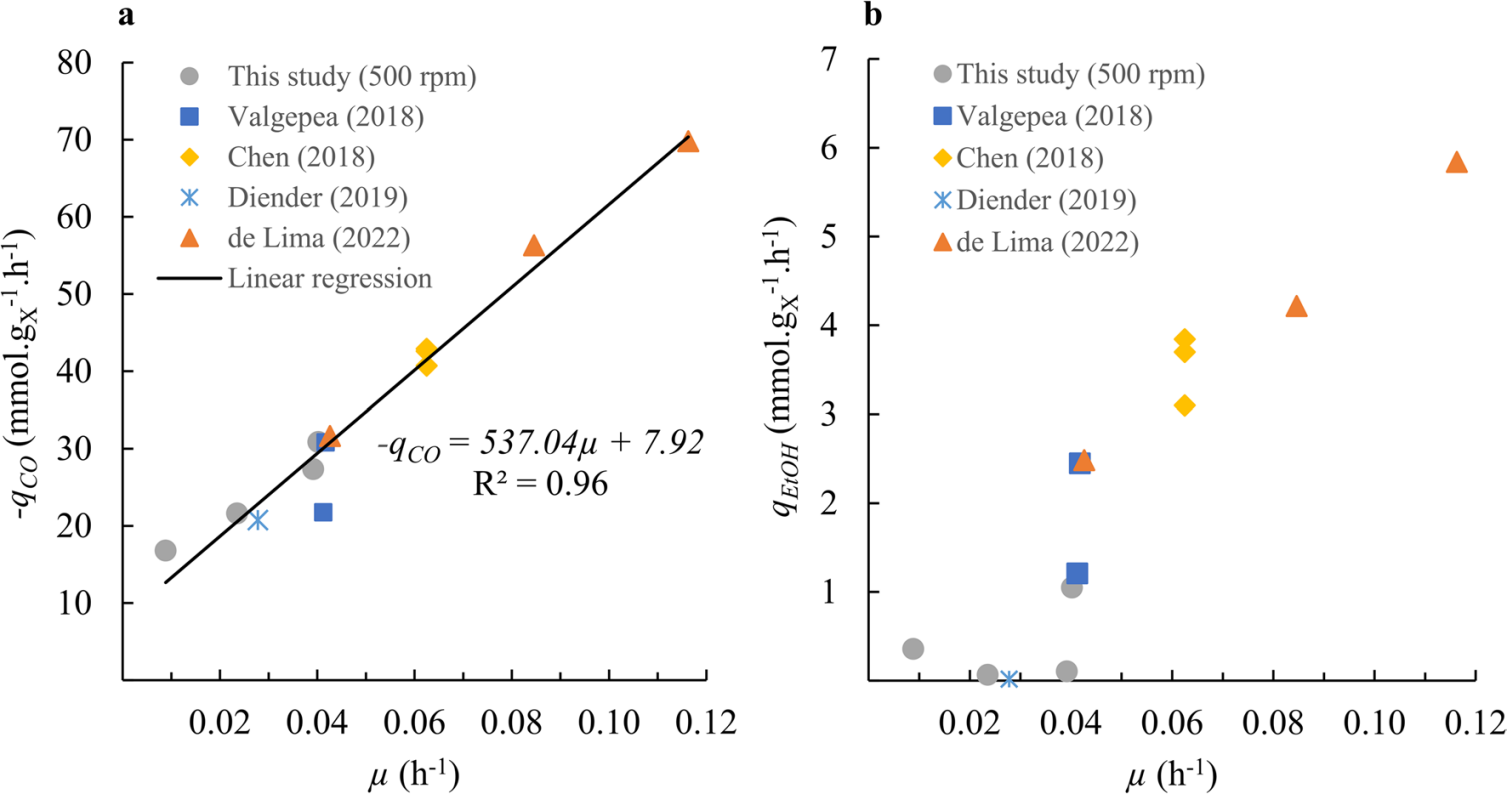
Biomass-specific rates of **a** CO and **b** EtOH as function of specific growth rate in continuous fermentations of *C. autoethanogenum* grown on CO

Figure 7 shows the dependence of *q*_CO_ and *q*_EtOH_ on *μ* for our experimental data at 500 rpm and diverse literature studies, for continuous CO fermentations by *C. autoethanogenum* under diverse cultivation conditions that avoided formate production. No *q*_CO_ data were obtained at 100 rpm due to equipment malfunctioning. While our experiments and those reported by Diender et al. (2019) used media containing yeast extract and at pH 5.9 and 6.2, respectively, other studies used chemically defined medium at pH 5 (Chen et al. 2018; Valgepea et al. 2018; de Lima et al. 2022). All studies used a stirred tank reactor, except Chen et al. (2018) who used a bubble column reactor. Furthermore, gas flow, CO composition in inlet gas, and agitation rate (if applicable) vary widely between the different studies analysed here.

Still, *q*_CO_ clearly correlates with *µ*, largely according to the Pirt equation (Heijnen and Kleerebezem 2010):

8 $$- q_{CO} = \frac{1}{{Y_{x/CO}^{\max } }}\mu + m_{CO}$$

However, the obtained maximum yield of biomass on substrate, $Y_{x/CO}^{max} = 0.0{76}\, \pm \,0.00{\text{5 mol}}_{{\text{X}}} {\text{ mol}}_{{{\text{CO}}}}^{ - 1}$, and maintenance coefficient, *m*_*CO*_ = 0.20 ± 0.05 mol_CO_ mol_X_^−1^ h^−1^, from this figure are merely apparent values because the undissociated acetic acid concentration results influence the amount of ATP required for maintenance (Valgepea et al. 2017), and different amounts of CO are consumed depending on the catabolic product. de Lima et al. (2022) claimed that increasing the growth rate increases *q*_EtOH_ and/or the volume-specific productivity of EtOH (*r*_EtOH_), but our experiments did not confirm this for *q*_EtOH_ (Fig. 7b) or *r*_EtOH_ (not shown). de Lima et al. (2022) increased the agitation rate for faster growing rates experiments to obtain equivalent biomass concentrations between the different steady states, which also resulted in higher total acetate concentrations and, consequently, higher undissociated acetic acid concentrations than in our experiments (Fig. 6). As discussed previously, higher acetic acid concentrations drive metabolic shifts towards ethanol production. In our study, the fermentations were cultivated at pH 5.9, which, using acetic acid p*K*_a_ of 4.77, results in a fraction of 6.7% undissociated acetic acid over total acetate, whereas this fraction increases to 36% at pH 5, which was used by de Lima et al. (2022). For a fixed growth rate, there are other experimental conditions that can affect ethanol production. Namely, pH and CO mass transfer rate (which are directly linked to acetic acid concentration) and media composition (including yeast extract concentration) have a major relevance.

Yeast extract has been reported to provide the required trace nutrients for the structural integrity of CO-fermenting *Clostridium* bacteria (Barik et al. 1988), besides being an important nitrogen source for the microorganisms and having a positive effect in lag phase duration (Diender et al. 2016). Nonetheless, lowering yeast extract concentration in the feed medium has been shown to result in enhanced production of more reduced products (such as ethanol) (Vega et al. 1989; Klasson et al. 1992; Abubackar et al. 2011). This could explain some differences regarding ethanol productivity with faster growth rates between our experimental results and the discussed literature.

### Implications for ethanol yield, titre, and production rate

By imposing different agitation rates in steady-state fermentations, we showed that insufficient mass transfer rate results in the excretion of the intermediate metabolite formate, while increasing mass transfer rates results in higher acetate and ethanol titres, yields, and productivities. Our study did not focus on maximizing ethanol concentration. Nevertheless, based on our results, we conclude that to obtain commercially interesting ethanol concentrations, much higher CO mass transfer rates will be needed (to provide sufficient carbon and reducing equivalents), while the dilution rate should still be modest to prevent dilution. We hypothesise that the extracellular undissociated acetic acid concentration is the crucial variable determining ethanol yield and production rate. In fact, our results strongly suggest that, by increasing the extracellular undissociated acetic acid concentration, *C. autoethanogenum* shifts CO metabolism towards ethanol production as a strategy to cope with acetic acid inhibition. A high yield of ethanol on CO requires > 20 mmol/L undissociated acetic acid, which can be obtained by (a combination of) high CO transfer rate, low pH, low dilution rate, and external acetate addition. Our research extends beyond previous studies on *C. ljungdahlii* (Richter et al. 2016; Schulz et al. 2023) by investigating the strain *C. autoethanogenum*, showcasing the impact of acetic acid inhibition across CO-fermenting acetogen species. These outcomes could shed light on strategies for industrial process operations and to drive metabolic shifts towards solventogenesis in CO fermentations. Additionally, acetic acid inhibition should be included in stoichiometric and kinetic models for accurate prediction of CO uptake rate, product distribution, yields, and titres.

## Data Availability

The Excel file with the steady-state source data underlying the manuscript figures and tables is available at 4TU.ResearchData at 10.4121/22285024.
